# Etymologia: Reproduction Number

**DOI:** 10.3201/eid2908.221445

**Published:** 2023-08

**Authors:** Vijay Sharma, Rajnish Sharma, Balbir B. Singh

**Affiliations:** Guru Angad Dev Veterinary and Animal Sciences University, Punjab, India

**Keywords:** etymologia, reproduction number, basic reproductive number, R_0_, effective reproductive number, R_t_, Z_0_, epidemics, new cases, infections, epidemiology, mathematical epidemiology, disease susceptibility, malaria, George Macdonald

**Reproduction Number [′ṝe-prə-′dak-shən ′nəm-bər]**

The basic reproduction number (R_0_, pronounced R naught) is derived from demography terminology used to estimate the overall population reproduction rate. R_0_ is an essential metric in the study of epidemics (Figure). This value measures the estimated number of new cases of an infection caused by an infectious person in a population of disease-susceptible person.

**Figure:**
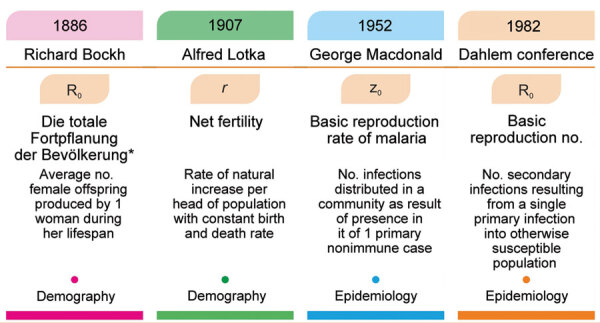
History and concept of basic reproduction number (R_0_). *The total reproduction of the population.

The effective reproduction number (R_t_) is similar to R_0_, but R_t_ measures the number of persons infected by infectious person when some portion of the population has already been infected. This idea can be traced back to the work performed by Richard Bockh, Alfred Lotka and others.

A modern application of R_0_ in epidemiology was reported in 1952 when George Macdonald constructed population models about the spread of malaria. Macdonald used the notation Z_0_ instead of R_0_ to differentiate it from the preceding demography terminology*.* The notation R_0_ was adopted instead of Z_0_ during the Dahlem conference in 1982.
